# Pulse contour analysis after normothermic cardiopulmonary bypass in cardiac surgery patients

**DOI:** 10.1186/cc3903

**Published:** 2005-11-04

**Authors:** Michael Sander, Christian von Heymann, Achim Foer, Vera von Dossow, Joachim Grosse, Simon Dushe, Wolfgang F Konertz, Claudia D Spies

**Affiliations:** 1 Department of Anesthesiology and Intensive Care Medicine, University Hospital Charité, Campus Charité Mitte, University Medicine, Schumannstrasse 20/21, 10098 Berlin, Germany; 2 Department of Cardiovascular Surgery, University Hospital Charité, Campus Charité Mitte, University Medicine, Schumannstrasse 20/21, 10098, Berlin, Germany

## Abstract

**Introduction:**

Monitoring of the cardiac output by continuous arterial pulse contour (CO_PiCCOpulse_) analysis is a clinically validated procedure proved to be an alternative to the pulmonary artery catheter thermodilution cardiac output (CO_PACtherm_) in cardiac surgical patients. There is ongoing debate, however, of whether the CO_PiCCOpulse _is accurate after profound hemodynamic changes. The aim of this study was therefore to compare the CO_PiCCOpulse _after cardiopulmonary bypass (CPB) with a simultaneous measurement of the CO_PACtherm_.

**Methods:**

After ethical approval and written informed consent, data of 45 patients were analyzed during this prospective study. During coronary artery bypass graft surgery, the aortic transpulmonary thermodilution cardiac output (CO_PiCCOtherm_) and the CO_PACtherm _were determined in all patients. Prior to surgery, the CO_PiCCOpulse _was calibrated by triple transpulmonary thermodilution measurement of the CO_PiCCOtherm_. After termination of CPB, the CO_PiCCOpulse _was documented. Both CO_PACtherm _and CO_PiCCOtherm _were also simultaneously determined and documented.

**Results:**

Regression analysis between CO_PACtherm _and CO_PiCCOtherm _prior to CPB showed a correlation coefficient of 0.95 (*P *< 0.001), and after CPB showed a correlation coefficient of 0.82 (*P *< 0.001). Bland-Altman analysis showed a mean bias and limits of agreement of 0.0 l/minute and -1.4 to +1.4 l/minute prior to CPB and of 0.3 l/minute and -1.9 to +2.5 l/minute after CPB, respectively. Regression analysis of CO_PiCCOpulse _versus CO_PiCCOtherm _and of CO_PiCCOpulse _versus CO_PACtherm _after CPB showed a correlation coefficient of 0.67 (*P *< 0.001) and 0.63 (*P *< 0.001), respectively. Bland-Altman analysis showed a mean bias and limits of agreement of -1.1 l/minute and -1.9 to +4.1 l/minute versus -1.4 l/minute and -4.8 to +2.0 l/minute, respectively.

**Conclusion:**

We observed an excellent correlation of CO_PiCCOtherm _and CO_PACtherm _measurement prior to CPB. Pulse contour analysis did not yield reliable results with acceptable accuracy and limits of agreement under difficult conditions after weaning from CPB in cardiac surgical patients. The pulse contour analysis thus should be re-calibrated as soon as possible, to prevent false therapeutic consequences.

## Introduction

Measurement of cardiac output (CO) is widely used in cardiac surgical patients. Over recent decades the main device for determination of CO has been the pulmonary artery catheter (PAC). The use of the PAC has been decreasing over recent years in surgical and cardiac surgical patients, however, as the benefit of guiding therapy with this device is unclear and the use of the PAC might even lead to increased morbidity, as shown in one large trial [1]. Other randomized studies indicate no clear evidence of benefit or harm by managing critically ill patients with a PAC [2,3].

Aortic transpulmonary thermodilution, a less invasive technique for determination of the CO, was therefore developed and has gained increasing acceptance in clinical practice [4-6]. Only an arterial line and a central venous line are needed to determine the CO by this method [7]. Several investigators found a good correlation between these two methods of CO determination [4-6,8]. The device mostly used also offers continuous CO determination by arterial pulse contour analysis. Stroke volume calculation and CO calculation by pulse contour analysis was developed years ago and underwent several methodological improvements of the algorithm [9,10]. Monitoring of the CO by continuous arterial pulse contour analysis (CO_PiCCOpulse_) is a widely used and clinically validated procedure proved to be an alternative to the pulmonary artery catheter thermodilution CO (CO_PACtherm_) in cardiac surgical patients [4,11]. Pulse contour monitoring demonstrated accuracy comparable with that of pulmonary artery thermodilution using a clearly less invasive approach [5,11,12]. There is ongoing debate, however, of whether the CO_PiCCOpulse _is accurate and reliable after profound changes of the hemodynamic situation, such as after cardiopulmonary bypass (CPB) [4,13].

The aim of this study was therefore to compare the bias and the limits of agreement (two standard deviations) of the CO_PiCCOpulse _after CPB, with a simultaneous measurement of the CO_PACtherm_, as the gold standard of CO measurement.

## Materials and methods

### Patients

Following ethical committee approval and written informed consent, 50 patients were considered eligible for this clinical trial from February to November 2004. The inclusion criteria were age >18 and <75 years, and elective coronary artery bypass graft surgery. The exclusion criteria were withdrawal of consent, valve pathologies, a left ventricular ejection fraction <40% and symptomatic peripheral artery stenosis.

### Perioperative management

Oral premedication was 0.1 mg/kg midazolam. In all patients a femoral artery was cannulated with a 4-Fr cannula (Pulsiocath; Pulsion Medical AG, Munich, Germany) prior to induction of anesthesia. A central venous catheter and a pulmonary artery catheter (Thermodilution Catheter; Arrow, Reading, PA, USA) were inserted via the right internal jugular vein.

General anesthesia was induced with etomidate (0.2 mg/kg), 5 μg/kg fentanyl and 0.1 mg/kg pancuronium. Maintenance was with infusion of 5–10 μg/kg per hour fentanyl, boluses of 0.1 mg/kg midazolam, 0.03 mg/kg pancuronium and 0.6–1% end-tidal isofluorane. All patients were ventilated with an oxygen–air mixture (inspiratory oxygen fraction, 0.5) to maintain an end-tidal partial pressure of carbon dioxide of 35–45 mmHg. The CPB technique was normothermic using intermittent antegrade warm blood cardioplegia as described by Calafiore and colleagues [14]. Transfusion management was performed according to our standard operating procedure [15]. The durations of anesthesia, surgery and aortic occlusion and the number of coronary artery bypass grafts were recorded.

### Determination of cardiac output

Prior to CPB, the CO_PiCCOtherm _and the CO_PACtherm _were determined immediately after sternotomy under stable hemodynamic conditions.

All volume substitution was stopped during the measurements. The CO_PACtherm _and the CO_PiCCOtherm _were measured by triple injection of 10 ml iced isotone sodium chloride solution into the central venous line of the PAC. The CO_PACtherm _and the CO_PiCCOtherm _were calculated by commercially available monitors (CCO module, Solar 8000; Marquette Hellige, Freiburg, Germany; and PiCCO CCO monitor; Pulsion Medical AG). In case of a deviation >10% of a measurement, five measurements were performed and the highest and lowest were rejected. The CO_PiCCOpulse _measurement was automatically calibrated by the CO_PiCCOtherm _measurement. The CO_PACtherm _and the CO_PiCCOtherm _measurements were carried out simultaneously.

The measurement after CPB was carried out 15 minutes after decanulation of the aorta. The prerequisite for this measurement was an optimized preload and stable hemodynamic condition with no damping of the arterial pressure line, which could be achieved in all patients. At this time the CO_PiCCOpulse _was documented. Simultaneously, the CO_PiCCOtherm _and CO_PACtherm _were determined by thermodilution measurement as already described.

### Statistical analysis

All data are expressed as the mean and standard error of the mean. Statistical analysis was performed by linear regression analysis. The bias and limits of agreement (LOA) (two standard deviations) were assessed according to the method described by Bland and Altman [16]. All numerical calculations were carried out with SPSS for WINDOWS (release 11.5.1, ©1989–2002; SPSS Inc, Chicago, IL, USA).

## Results

Anesthesia and surgery were uncomplicated in all patients analyzed during this study. Five patients had to be excluded due to their impossibility to achieve a valid CO_PACtherm _or CO_PiCCOtherm _measurement. Therefore, 45 patients remained in the study for analysis. Basic patient characteristics are presented in Table 1. Hemodynamic data are presented in Table 2. The heart rate, CO_PACtherm _and CO_PiCCOtherm _increased significantly compared with the pre-CPB values. The systemic vascular resistance decreased significantly compared with the baseline measurement.

Prior to CPB, the regression analysis between the CO_PACtherm _and CO_PiCCOtherm _measurements showed an excellent correlation, with a correlation coefficient of 0.95 (*P *< 0.001). Bland–Altman analysis showed a mean bias and LOA of 0.0 l/minute and -1.4 to +1.4 l/minute. The regression analysis after CPB also showed a good correlation between the CO_PACtherm _and the CO_PiCCOtherm_, with a correlation coefficient of 0.82 (*P *< 0.001). The Bland–Altman analysis after CPB showed a mean bias and a precision of 0.3 l/minute and -1.9 to +2.5 l/minute.

Comparison of CO_PiCCOpulse _versus CO_PiCCOtherm _and of CO_PiCCOpulse _versus CO_PACtherm _showed only a fair correlation after CPB, with a correlation coefficient of 0.67 (*P *< 0.001) and 0.63 (*P *< 0.001), respectively. Bland–Altman analysis showed a mean bias and LOA of -1.1 l/minute and -1.9 to +4.1 l/minute versus -1.4 l/minute and -4.8 to +2.0 l/minute, respectively.

## Discussion

The main finding of this study is that the CO measured by pulse contour analysis was considerably different compared with the CO_PiCCOtherm _and the CO_PACtherm_. The CO_PiCCOtherm _and CO_PACtherm _measurements correlated well before and after CPB, indicating that CO measurement by pulse contour analysis needs to be recalibrated after CPB to achieve valid results.

Pulse contour analysis CO has been shown previously to serve as a valid and cost-effective device for CO determination after calibration [17]. In our study we investigated the validity of continuous CO measurement by pulse contour analysis after CPB. The main advantage of CO_PiCCOpulse _measurement after CPB would be the fast determination of CO. As soon as pulsatile flow is restored, the algorithm of the CO monitor automatically starts determination of the CO by continuous pulse contour analysis. Therefore, during a period when the anesthetist's full attention is focused on vasoactive and volume therapy necessary for successful weaning from CPB, a fast and continuous approach such as continuous pulse contour analysis might be much more practical than time-consuming intermittent thermodilution techniques for determination of CO. However, these advantages would only apply if the obtained data are valid.

The initial calibration of the CO_PiCCOpulse _measurement was performed by aortic transpulmonary CO determination prior to CPB. We found an excellent correlation between the CO_PiCCOtherm _and the CO_PACtherm _measurements. This correlation has been described by previous investigators [12]. After CPB the correlation remained good, but Bland-Altman analysis revealed a trend for the CO_PiCCOtherm _to slightly underestimate the CO, with increased LOA compared with the measurements prior to CPB. As we do not know the 'true' CO, it is speculative which CO measurement estimates more precisely the 'true' CO. An explanation for the greater scatter between the two CO measurements after CPB compared with the measurements prior to CPB might be an influx of cold blood. This cold blood might be derived from compartments, which might be hypoperfused during CPB and reperfused in the period after CPB as suggested by previous investigators [4,18]. Even though we performed normothermic CPB management, patients tended to display a slight decrease of their body temperature, worsening the signal-to-noise ratio of the thermal indicator used for determination of the CO by these methods. Better results in this setting might be achieved using an indicator independent from thermal signals. Given the increased LOA of the CO_PiCCOtherm _measurement, therefore, the calibration of the pulse contour analysis with a thermal indicator might be less than ideal in this period and should be repeated early after surgery.

After CPB, the pulse contour CO showed marked differences compared with the CO_PiCCOtherm _and CO_PACtherm _measurements. The CO_PiCCOpulse _measurement systematically underestimated the CO determined by the other two methods. This has been described previously [4]. In our investigation the CO and the heart rate increased significantly after CPB. We also observed a significant decrease in systemic vascular resistance after CPB. Differences between pulse contour CO and thermodilution CO measurements in patients with significant changes of the systemic vascular resistance [13] have already been established in previous investigations. Further studies are therefore needed, addressing also the performance of newly developed pulse contour devices that do not include an independent technique for calibration under difficult clinical settings, such as after CPB.

The fact that we failed to determine the CO by a method independent of thermal signals such as echocardiographic or lithium dilution measurement of the CO to validate the thermal dilution measurement [19,20] is a shortcoming of our study. Bearing in mind, however, that we did find an excellent correlation prior to CPB and a good correlation after CPB for the two thermodilution measurements, we believe that the thermodilution methods represent a reliable estimation of the 'true' CO in clinical practice. In case of severe hemodynamic instability after CPB, indicated by the CO_PiCCOpulse_, CO_PiCCOtherm_, CO_PACtherm _or other clinical parameters, echocardiography should be used to guide therapy as suggested previously [21]. It has been established formerly that pulse contour analysis CO is a valid and cost-effective device for CO determination after calibration. Another limitation is that the design of our study does not allow for an ultimate demonstration of a causal relationship between CPB and lack of agreement. However, a number of studies show that pulse contour analysis is valid for at least some hours if there are no severe changes in hemodynamics. The mean time between sternotomy and the start of CPB is about 60 minutes. We therefore think it is reasonable to assume that CPB is mainly responsible for the inaccuracy of the post-CPB pulse contour analysis observed in our study.

## Conclusion

In conclusion, we observed an excellent correlation of CO_PiCCOtherm _and CO_PACtherm _measurement prior to CPB. Our study could not prove pulse contour analysis with a modified Wesseling algorithm used in this study to be a method yielding reliable results with excellent accuracy and limits of agreement under difficult conditions after CPB in cardiac surgical patients. Hence, due to the broad distribution and the underestimation of the CO after CPB, the use of the uncalibrated continuous pulse contour cardiac output cannot be recommended after weaning from CPB. A re-calibration in this setting is essential.

## Key messages

• 	We observed an excellent correlation of CO_PiCCOtherm _and CO_PACtherm _measurement prior to CPB.

• 	Our study could not prove pulse contour analysis with a modified Wesseling algorithm to be a method yielding reliable results under difficult conditions after CPB in cardiac surgical patients.

• 	Due to the broad distribution and the underestimation of the CO after CPB, the use of the uncalibrated continuous pulse contour cardiac output cannot be recommended after weaning from CPB.

## Abbreviations

CO = cardiac output; CO_PACtherm _= pulmonary artery catheter thermodilution cardiac output; CO_PiCCOpulse _= continuous arterial pulse contour analysis cardiac output; CO_PiCCOtherm _= aortic transpulmonary thermodilution cardiac output; CPB = cardiopulmonary bypass; LOA = limits of agreement; PAC = pulmonary artery catheter.

## Competing interests

The authors declare that they have no competing interests.

## Authors' contributions

MS and CvH prepared the manuscript, carried out the cardiac output measurements, conceived of the study and performed the statistical analysis. AF, JG and VvD helped with the recruitment of the patients and the drafting of the manuscript. SD and WFK participated in the study design and helped with the recruitment of patients. CS drafted the manuscript, helped with the study design and coordination. All authors read and approved the final manuscript.

## Figures and Tables

**Figure 1:**
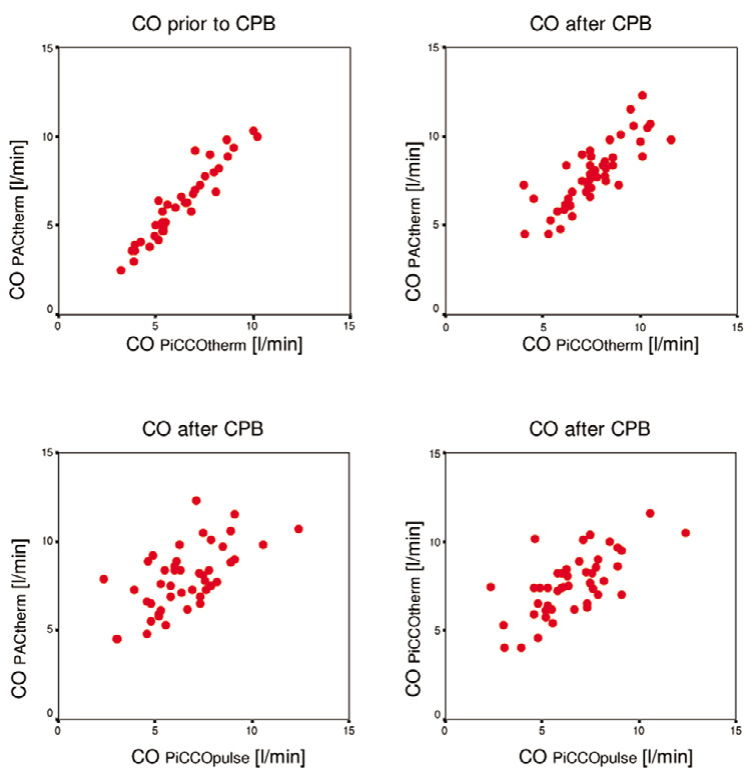
Regression analysis of pulmonary artery catheter thermodilution cardiac output (CO_PACtherm_) versus aortic transpulmonary thermodilution cardiac output (CO_PiCCOtherm_) prior to and after cardiopulmonary bypass (CPB), and regression analysis of continuous arterial pulse contour analysis cardiac output (CO_PiCCOpulse_) versus CO_PiCCOtherm _and versus CO_PACtherm _after CPB.

**Figure 2:**
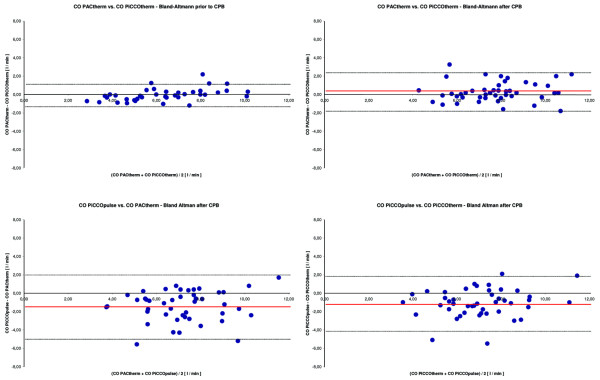
Bland-Altman plot of pulmonary artery catheter thermodilution cardiac output (CO_PACtherm_) versus aortic transpulmonary thermodilution cardiac output (CO_PiCCOtherm_) prior to and after cardiopulmonary bypass (CPB), and Bland-Altman plot of continuous arterial pulse contour analysis cardiac output (CO_PiCCOpulse_) versus CO_PiCCOtherm _and versus CO_PACtherm _after CPB. CO, cardiac output.

**Table 1:** Patient characteristics

	Mean	Standard error of the mean
Age (years)	62	1
Height (m)	1.77	0.01
Body weight (kg)	91	2
Body mass index (kg/m^2^)	29.1	0.6
Number of grafts (*n*)	3	0
Duration of anesthesia (minutes)	314	7
Duration of surgery (minutes)	201	6
Temperature prior to cardiopulmonary bypass (°C)	35.2	0.1
Temperature after cardiopulmonary bypass (°C)	36.1	0.1
Cardiopulmonary bypass time (minutes)	71	3
Aortic clamping time (minutes)	44	2

**Table 2:** Hemodynamic data

	Mean	Standard error of the mean
Heart rate prior to CPB (l/minute)	69	3
Heart rate after CPB (l/minute)	81*	2
Mean arterial pressure prior to CPB (mmHg)	70	2
Mean arterial pressure after CPB (mmHg)	73	2
Central venous pressure prior to CPB (mmHg)	9	1
Central venous pressure after CPB (mmHg)	11	1
Mean pulmonary arterial pressure prior to CPB (mmHg)	21	1
Mean pulmonary arterial pressure after CPB (mmHg)	20	1
Pulmonary wedge pressure prior to CPB (mmHg)	11	1
Pulmonary wedge pressure after CPB (mmHg)	12	1
Systemic vascular resistance prior to CPB (dyn/s per cm)	861	53
Systemic vascular resistance after CPB (dyn/s per cm)	727*	47
Pulmonary vascular resistance prior to CPB (dyn/s per cm)	115	10
Pulmonary vascular resistance after CPB (dyn/s per cm)	93	8
CO_PACtherm _prior to CPB (l/minute)	6.2	0.4
CO_PACtherm _after CPB (l/minute)	7.9*	0.3
CO_PiCCOtherm _prior to CPB (l/minute)	6.2	0.3
CO_PiCCOtherm _after CPB (l/minute)	7.6*	0.3
CO_PiCCOpulse _after CPB (l/minute)	6.5	0.3

CPB, cardiopulmonary bypass. CO_PACtherm_, pulmonary artery catheter thermodilution cardiac output; CO_PiCCOtherm_, aortic transpulmonary thermodilution cardiac output; CO_PiCCOpulse_, continuous arterial pulse contour analysis cardiac output.

*P < 0.05 compared with baseline.
